# Optimizing work in community pharmacy: What preferences do community pharmacists and pharmacy technicians have for a better allocation of daily activities?

**DOI:** 10.1016/j.rcsop.2024.100549

**Published:** 2024-12-11

**Authors:** Jean-Didier Bardet, Jérôme Combe, Arnaud Tanty, Perrine Louvier, Mathieu Granjon, Benoît Allenet

**Affiliations:** a Equipe ThEMAS, Univ. Grenoble Alpes, CNRS, Grenoble INP, CHU Grenoble Alpes, TIMC-IMAG, 38000 Grenoble, France; b UFR Pharmacie, Univ. Grenoble Alpes, Grenoble, France; c Pharmacy, CHU Grenoble Alpes, Grenoble, France

## Abstract

**Objective:**

Pharmacy work encompasses two main streams. These are logistic flow (the supply and distribution of healthcare products) and pharmaceutical flow (the dispensing and provision of pharmacy services). The pharmaceutical flow has increased significantly with the introduction of reimbursed services such as Rapid Diagnostic Tests, chronic disease screening, minor ailment prescriptions, vaccine prescription and administration, and medication reviews. The implementation of new pharmacy services requires efficiency. The main objective of the survey presented here was to determine the preferences of community pharmacists and pharmacy technicians in relation to the assignment of tasks within the community pharmacy team.

**Method:**

The survey, which used the Best-Worst Scaling (BWS) method, presented 13 daily pharmacy activities to community pharmacists (CPs) and pharmacy technicians (PTs). Descriptive statistics and Bayesian logistic regression were used to analyse the data.

**Key findings:**

The results indicate that medication dispensing is a shared activity between CPs and PTs, for which the latter already have partial autonomy. Management of nursing home orders and supplies tends to be assigned to pharmacy technicians, whereas clinical pharmacy services such as prescription renewal, medication reviews, and counselling are considered to be more within the CPs' domain. CPs would readily delegate tasks like screening procedures to PTs. PTs express interest in minor ailment prescribing, a responsibility CPs are not yet ready to entrust to them.

**Conclusions:**

Delegating logistical activities to pharmacy technicians could enable community pharmacists to focus on specialized pharmaceutical care, thereby improving the efficiency and quality of the services offered to patients. However, the reorganization of tasks should not only be implemented from a logistical standpoint since pharmacy technicians also share an interest in pharmaceutical care.

## 1 Introduction

Pharmacy work can be broadly categorized into two main streams: the logistic flow, which involves the supply and distribution of healthcare products, and the pharmaceutical flow, which focuses on dispensing^1^ and providing pharmacy services. In recent years, the pharmaceutical flow has been experiencing a significant expansion, driven by the introduction of a variety of reimbursed pharmacy services. In France, for instance, more than a dozen new pharmacy services have been introduced since 2009; including Rapid Diagnostic Tests (RDTs), chronic disease screening, minor ailments prescribing, the prescription and administration of vaccines to individuals aged 11 and over, medication and disease counselling, and medication reviews. Further pharmacy services, including the renewal of chronic treatments for a 3-month period are also anticipated in the near future.^2^

This shift towards expanded pharmacy services occurs in a context of reduced access to direct medical care,^3^ and increasing demands on pharmacy staff.^4^ The need to provide high-quality pharmaceutical care while managing limited resources highlights the importance of task delegation to enhance efficiency.^5^ Enhanced pharmacy services vary significantly in their complexity of implementation. Pharmacy services which are focused on therapeutic optimisation, such as medication counselling, medication reviews, and chronic medication renewals are more complex services due to the significant time commitment required from community pharmacists (CPs).^6^ In contrast, services like minor ailment prescribing and RDTs are easier to integrate into daily pharmacy operations, as they address immediate patient needs and are relatively straightforward to implement.

The evolution of pharmacy practice also underscores the growing importance of aligning pharmacy technicians' (PTs) motivations and training with their expanding roles.^7^ A recent reform aims to harmonize the different levels of education within the European Union. This reform presents an opportunity to modernise the initial training programmes for pharmacy technicians (PTs) in France. Notably, 20 % of the courses now on offer must be taught by academics from the faculties of pharmacy,^8^ highlighting the increasing emphasis on bridging technical and clinical competencies. PTs play a vital role in both the logistic and pharmaceutical workflows: managing orders and dispensing healthcare products, including OTC medications, under the supervision of CPs. The COVID-19 pandemic has further accelerated the transformation of PTs roles. In response to the need for widespread vaccination, they contributed to vaccination efforts. A law passed in 2023 to facilitate access to healthcare, has aimed to institutionalize the transitional provisions for administering vaccines by PTs under CP's supervision. This is pending the implementing decrees.^2^

Thus, the professions constituting the community pharmacy team are greatly evolving. The demand from the population for pharmaceutical care is on the increase amid limited human resources. In this context, how should CP time for activities requiring pharmaceutical expertise be allocated? How can one enhance the efficiency of the different pharmacy work processes to ensure consistent access to quality pharmaceutical care as well as its continuity? One potential approach would be to streamline the activities, for example, by adopting synchronized dispensing, as practiced in North America.^9^ Another one would be to optimize the assignment of tasks within the pharmacy team. This survey focuses on the latter approach, investigating the preferences of CPs and PTs regarding their daily activities.

## 2 Method

### 2.1 Best-worst scaling method

The survey was based on the Best Worst Scaling (BWS) method,^10^^,^^11^ a discrete choice of method aimed at capturing individual preferences. The concept of preferences in economics is based on measuring utility to understand individual choices. Utility refers to the satisfaction an individual derives, particularly from engaging in activities. In our study, this utility is determined by the perception of the skills required, the impact on the pharmacy, and the personal satisfaction gained from performing these tasks.

The BWS method relies on the idea that individuals select, from a set of options, the pair that represents the greatest variation in preference for them. They are, therefore, choosing between the two activities that differ the most in terms of their alignment with their skills, their role in the pharmacy, and their ability to maximize their sense of contribution within it.

A BWS survey involves a questionnaire which presented the respondents with series of different scenarios. Each scenario offers a fixed number of items, referred to as attributes, and respondents must indicate which attribute is best and which is worst for them. Repetition of the scenarios enables the ranking and weighting of attributes. By capitalising on respondents, selecting the pair that reflects the maximum difference in preference or importance to them, BWS offers methodological advantages over other ranking methods, allowing for a more discriminatory ranking of attributes. While traditionally used with ‘best’ and ‘worst’ choice instructions for respondents, this method can be applied to any situation where a gap in preferences needs to be measured.

### 2.2 Survey distribution and inclusion criteria

The questionnaire was distributed electronically from February 2nd to February 23rd 2023, via various channels, including the Faculty of Pharmacy at the University Grenoble Alpes emailing lists (internship supervisors and former pharmacy students) and social media (Facebook®, X®). These professionals received an email describing the purpose of the study along with a consent form and inviting them to complete the survey online. To be included, the respondents had to be currently practising in France and be either a PT in a community pharmacy or a CP.

### 2.3 BWS questionnaire

Before completing the questionnaire, respondents were provided with a brief description of the study objectives.

A total of 13 activities (Table 1) performed in daily pharmacy practice were identified, based on the framework for community pharmacy practice used by the French Society of Clinical Pharmacy (SFPC),^12^ the French framework for Quality Assurance in Community Pharmacy,^13^ and the regulatory provisions regarding new pharmacy services. Each identified pharmacy activity corresponded to one attribute of the BWS. The most optimal design was defined by using the BWSTool package version 1.2.0 (design 27, seed = 2000) of R software version 4.2.1, thus ensuring both balance and orthogonality.

**Table 1:** Attributes (pharmacy activities) tested in the BWS (by flow and in alphabetical order).

Attributes	Definitions
*Pharmaceutical flow*	
Drug dispensing in autonomy	Dispensing healthcare products associated with medical prescriptions and on patient's request for Over-The-Counter pharmaceuticals
Medication counselling	Conducting counselling interviews for anticoagulants, asthma, and oral chemotherapy with patients to improve disease understanding, adherence to treatments, and reduce the risk of iatrogenic issues
Medication review	Conducting patient interviews, pharmaceutical analysis, reporting to the physician, and advisory and adherence follow-up interviews
Prescription for minor ailments^a^	Dispensing on-prescription medicines for the management of cystitis, sore throat, chickenpox, and allergic rhino-conjunctivitis
Renewal and dosage adjustment a	Renewal and dose adjustment for chronic treatments in patients with chronic diseases
Screening procedures	Conducting: capillary blood glucose tests to detect abnormal glucose levels as part of a diabetes prevention campaign; oropharyngeal diagnostic tests for Strep A, nasopharyngeal diagnostic tests for influenza and SARS-Cov2
Vaccine administration^a^	Administration of the 18 approved vaccines (Diphtheria, Tetanus, Poliomyelitis, Whooping Cough, Influenza, Human Papillomavirus, Measles, Mumps, Rubella, Hepatitis A, Hepatitis B, Meningococcal ACYW, Meningococcal B, Pneumococcus, Chickenpox, Shingles, Yellow Fever, Rabies) for individuals over 16 years old
Vaccine prescription	Prescribing vaccination renewals for the 18 approved vaccines (Diphtheria, Tetanus, Poliomyelitis, Whooping Cough, Influenza, Human Papillomavirus, Measles, Mumps, Rubella, Hepatitis A, Hepatitis B, Meningococcal ACYW, Meningococcal B, Pneumococcus, Chickenpox, Shingles, Yellow Fever, Rabies) for individuals over 16 years old

*Logistic flow*	
Daily order management	Receiving orders from wholesalers and laboratories, managing stocks, and merchandising
Purchasing management	Identifying the needs of the pharmacy, implementing a purchasing strategy, comparing and evaluating offers from potential suppliers, negotiating directly with suppliers, and establishing a pricing policy

*Mixt flow (logistic and pharmaceutical flow)*	
Nursing home supply management	Assessing the needs of the nursing homes, managing orders, tracking dispensations, and preparing doses for administration

*Management*	
Team management	Organizing work, defining schedules, and assigning tasks to pharmacy staff, participating in recruitments
Quality management	Leading the quality assurance process in the pharmacy

a These activities are not yet authorized for pharmacists and/or pharmacy technicians; some may exist under protocolized practices in collaborative practice structures; they may also be considered as likely developments in the short or medium term for community pharmacies.

The survey design included a total of 13 scenarios, each presenting 4 attributes for respondents to choose from. Each attribute is presented a total of 4 times in the entire survey; each attribute pair is presented once; and each attribute is presented once in position 1, 2, 3, or 4 in the entire survey. All participants were presented with the same set of 13 BWS tasks. The 13 activities were presented to respondents as outlined in Table 1, prior to responding to the BWS exercise.

**Fig. 1:**
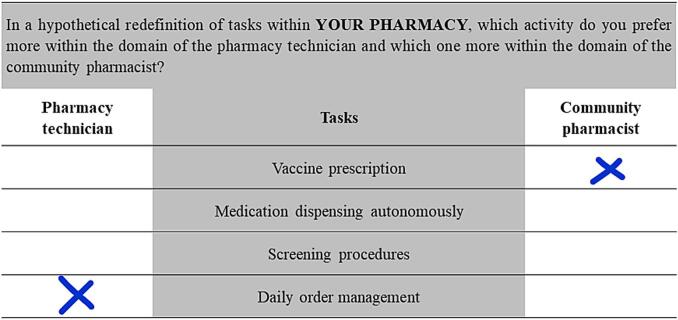
Example of a scenario presented for a respondent's choice, with suggested response - scenario 11.

In accordance with the BWS method, in this survey, respondents were asked to position themselves in each scenario by indicating which activity they consider belongs more to the domain of the PT or to that of the CP (Fig. 1).

Socio-demographic questions were placed at the end of the questionnaire. The socio-demographic data of the respondents collected included: age, gender, professional status, and certain characteristics of the respondent's community pharmacy (the administrative unit in France, the size of the city in which the pharmacy is located as well as the type of pharmacy).

### 2.4 Statistical analyses

Descriptive statistics were used to analyse the responses. The Qualitative variables are presented in both numbers and percentages. The Quantitative variables are described as means, median and standard deviations (SD), minimum, and maximum for continuous variables, depending on the distribution of the data. Regarding the preferences, weighted scores were calculated by Bayesian logistic regression using the BWSTool package version 1.2.0 (R software version 4.2.1; design 27, seed = 2000) for each activity and for the 3 types of respondent: (i) owner CPs (OCPs); (ii) employed CPs (ECPs) and (iii) PTs. The results are presented according to preference of the activities towards the PT (*b* > 0). The RAI (Relative Attractiveness Index) scores were calculated for the three populations. This approach allowed us to compare the relative attractiveness of the various attributes across the three populations, providing a normalized measure of their preferences. Differences in relative attribute were also explored.

## 3 Results

### 3.1 Sociodemographic characteristics of the respondents

A total of 181 respondents participated in the survey, including 97 (53.6 %) OCPs, 48 (26.5 %) ECPs and 36 (19.9 %) PTs. Among them, 70.2 % (*n* = 127) were female and the median age of the respondents was 39.9 years old (SD 10.8) (Table 2). The respondents mainly work in pharmacies which are located in municipalities of less than 10,000 inhabitants (*n* = 108; 57.8 %) and therefore in pharmacies which can be categorized as being rural (*n* = 69; 38.1 %) or neighbourhood pharmacies (*n* = 66; 36.5 %).

**Table 2:** Sociodemographic characteristics of participants and practice locations.

	*n* = 181*	
Participants		
*Owner community pharmacists*		
n	97	
female	66	(68,0 %)
mean age	45,2 yo	(SD = 10,8)
*Employed community pharmacists*		
n	48	
female	27	(56,3 %)
mean age	32,6 yo	(SD = 8,1)
*Pharmacy technicians*		
n	36	
female	34	(94,4 %)
mean age	35, 5 yo	(SD = 10,8)
Size of the town		
rural municipality	31	(16,6%)
<5000 inhabitants	36	(19,3 %)
5000 to 10,000 inhabitants	41	(21,9 %)
10,000 to 20,000 inhabitants	13	(7,0 %)
20,000 to 50,000 inhabitants	23	(12,3 %)
50,000 to 100,000 inhabitants	16	(8,6%)
100,000 to 200,000 inhabitants	13	(7,0 %)
>200,000 inhabitants	14	(7,5 %)
Type of Pharmacy		
Town (rural) pharmacy	69	(38,1 %)
City centre pharmacy	31	(17,1 %)
Neighbourhood pharmacy	66	(36,5 %)
Shopping Mall pharmacy	11	(6,1 %)
Tourist area pharmacy	4	(2,2 %)

### 3.2 Preferences for daily activities

A total of 2353 choice tests were analysed. For each professional group, the ranking of the activities according to whether these were considered to belong more to the PT domain or to the CP domain is presented in Table 3 (graphical presentation in Appendix A).

**Table 3:** Aggregated scores by population.

Attributes	Owner Pharmacists		Employed Pharmacists		Technicians	
	Coefficients	SD	Coefficients	SD	Coefficients	SD
Daily order management	1.31	0.09	1.63	0.14	1.38	0.15
Screening procedures	1.09	0.08	0.96	0.11	0.37	0.12
Nursing home supply management	0.93	0.08	0.81	0.11	0.76	0.13
Vaccine administration	0.62	0.08	0.50	0.11	0.08	0.12
Drug dispensing in autonomy	0.26	0.07	0.25	0.10	0.51	0.12
Quality management	−0.03	0.07	0.11	0.10	−0.50	0.12
Purchasing management	−0.05	0.07	0.35	0.10	0.08	0.12
Prescription for minor ailments	−0.16	0.07	−0.41	0.10	0.32	0.12
Vaccine prescription	−0.39	0.07	−0.51	0.11	−0.54	0.12
Team management	−0.66	0.08	−0.73	0.11	−0.60	0.12
Medication counselling	−0.78	0.08	−0.78	0.11	−0.65	0.12
Medication review	−0.94	0.08	−0.84	0.11	−0.41	0.12
Renewal and dosage adaptation for chronic patients	−1.10	0.08	−1.20	0.12	−0.66	0.12

### 3.3 Activities assigned preferably to PTs

For all 3 types of respondent, the TOP3 activities preferably assigned to PTs include: order management and the supply management of nursing homes. For the owners and salaried pharmacists, this TOP3 includes screening procedures while for the PTs, it includes the independent dispensing of health products.

### 3.4 Activities preferably assigned to CPs

All activities related to clinical pharmacy services are within the competency of CPs for all 3 types of respondents, which covers prescription renewal and dosage adjustment, medication review and medication counselling. The professional field of pharmacists also includes team management.

### 3.5 Significant differences in weight between respondents' preferences

**Fig. 2:**
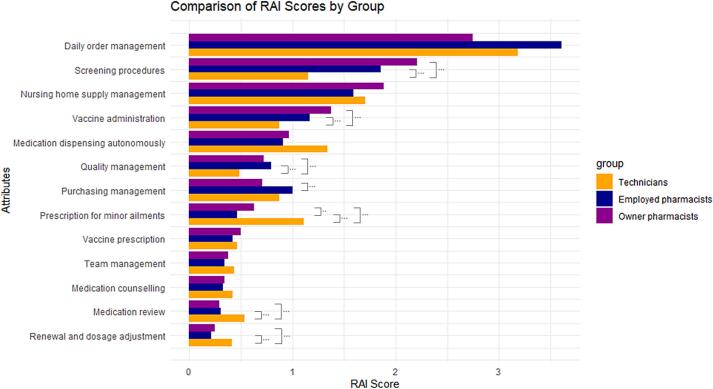
Preferences of community pharmacists and pharmacy technicians on daily activities.

According to the type of respondent, the weight of preferences varies significantly for 6 activities (Table 4). The relative importance of each activity in its assignment to technicians is presented in Fig. 2: a RAI score greater than 1 indicates a preference for the activity to be performed by PTs, while a RAI score below 1 indicates a preference for it to be performed by CPs.

**Table 4:** Comparison of Scores between the 3 Populations.

Attributes	Owner vs Employed Pharmacists		Owner Pharmacists vs Technicians		Employed Pharmacists vs Technicians	
	Z Stat	*p* Value	Z Stat	*p* Value	Z Stat	*p* Value
Vaccine administration	0.90	0.3657	3.81	0.0001***	2.63	0.0084***
Screening procedures	0.90	0.3656	4.99	0.0000***	3.62	0.0003***
Medication review	−0.75	0.4540	−3.68	0.0002***	−2.62	0.0088***
Drug dispensing in autonomy	0.10	0.9171	−1.74	0.0819	−1.63	0.1034
Prescription for minor ailments	1.99	0.0468**	−3.46	0.0005***	−4.63	0.0000***
Medication counselling	−0.02	0.9815	−0.91	0.3635	−0.78	0.4337
Nursing home supply management	0.88	0.3762	1.19	0.2358	0.34	0.7366
Daily order management	−1.95	0.0507	−0.40	0.6890	1.24	0.2134
Team management	0.50	0.6181	−0.43	0.6702	−0.78	0.4373
Quality management	−1.12	0.2608	3.33	0.0009***	3.85	0.0001***
Purchasing management	−3.12	0.0018***	−0.94	0.3473	1.68	0.0929
Vaccine prescription	0.93	0.3525	1.05	0.2953	0.18	0.8538
Renewal and dosage adaptation for chronic patients	0.68	0.4964	−2.92	0.0035***	−3.09	0.0020***

Significance levels: ** for *p* < 0.05, *** for *p* < 0.001

Pharmacists (OCPs and ECPs) have showed a significantly higher preference than PTs for being assigned screening procedures (*p* < 0.001), vaccine administration (p < 0.001) and quality assurance management (*p* < 0.001) to PTs.

Even if all the types of participants agree that medication review is assigned to CPs, the weight of the PTs' preferences are significantly higher than those of the pharmacists (OCPs and ECPs) where medication review (*p* < 0.001) and renewing and adjusting dosages for chronic patients (*p* < 0.001) are concerned.

A disagreement arises regarding the prescription of minor ailments: the technicians show a marked preference for this activity, whereas the pharmacists (OCPs and ECPs) consider it part of their own scope (*p* < 0.001). It is also noted that pharmacists' preference to consider it within their scope is more pronounced for ECPs (*p* < 0.05).

Regarding purchasing management, ECPs are more inclined to assign this activity to pharmacy technicians than OCPs (*p* < 0.001).

## 4 Discussion

This survey is unique as it has determined not only the preferences of CPs, both owners and employees, but also those of PTs, on the organisation and/or allocation of daily activities carried out by community pharmacy teams. It appears that 2 activities are clearly seen to be the responsibility of PTs: order management and supplies to nursing homes. In the same way, two types of activity are viewed as the responsibility of CPs: team management and clinical pharmacy services.

### 4.1 Limitations

The survey comprised 198 respondents. The average ages of the three categories of participants are lower compared to the available demographic data for these professions^4^^,^^14^: it is 4 years younger for OCPs, 12 years younger for ECPs, and 3 years younger for PTs. It is difficult to assess the impact of this difference on the responses. These differences are explained by the recruitment method.

While the number of respondents may seem limited compared to the number of professionals in practice, the BWS method offers the advantage of leveraging effects by submitting the same item to the respondents' choice several times since, in this survey, each activity was presented four times to each respondent.

Responding to a BWS experiment requires a cognitive effort from participants and may introduce redundancy, thus potentially limiting response rates. To minimize comprehension difficulties and cognitive effort, the labels used in this survey and the formulations chosen corresponded to everyday activities in pharmacy practice and were well understood by the respondents.

### 4.2 The pharmacy practice in France

The results must also be considered in light of the specific characteristics of community pharmacy practice in France. Community pharmacies are small structures, with an average of four employees.^14^ Nationally, half of the salaried positions are held by pharmacy technicians, and a quarter by salaried pharmacists. The remaining positions include cosmetic sales assistants or maintenance staff. Warehouse workers and administrative roles are rare.

In France, all medicines are packaged in unseparable units, so the pharmacy teams do not handle bulk or repackaging. Moreover, compounded preparations are outsourced. This is why PTs are already highly involved in the dispensing of healthcare products, with minimal supervision.

There is little international data regarding the evaluation of workflow and the time spent on various tasks by the different community pharmacy team members.^15^, ^16^, ^17^, ^18^, ^19^ The CP working time dedicated to administrative or non-professional/semi-professional tasks is estimated at between 30 and 50 % of their time,^15^, ^16^, ^17^, ^18^ and this amount appears to have been stable over the past 20 years.^15^^,^^17^ Focusing on CPs rather than the pharmacy team, this data seems consistent with what can be observed in the field in France.

### 4.3 *Re*-engineering workflow

How should we use the preferences of pharmacy team professionals to rethink and optimize daily workflow? The increase in pharmacy activities, both for triage and continuity of care, requires a reorganization of the workflow. The objective is not only to free up pharmacists' time for these new activities but also to evaluate how pharmacy technicians position themselves in relation to them. The allocation of tasks must consider both the type of activity and the complexity of patient care.

### 4.4 Support functions

As the pharmaceutical workflow is increasing due to the addition of new tasks, this survey shows that transferring logistics flow activities to PTs is the first avenue for improving the overall workflow in pharmacies.^5^^,^^7^In this survey, order management is unequivocally the responsibility of PTs for all the respondents, and that CPs prioritize this task for them in particular Depending on the studies, this logistical aspect can be estimated to occupy between 20 and 30 % of CP time.^15^^,^^16^ In French pharmacies, this activity is usually shared between PTs and CPs – especially ECPs – whether it concerns order reception or merchandising. However, these last two activities only pertain to the final part of the supply chain management of a community pharmacy. Although this survey reveals no clear preference for transferring purchasing management to PTs, a significant difference emerges among ECPs, who are more inclined than OCPs to delegate this responsibility to PTs. In practice, when purchasing management is delegated by OCPs, there is little distinction between whether it is managed by an ECP or a PT. Thus, from the perspective of an ECP aiming to allocate more time to patient care, entrusting this responsibility to a PT appears to be a logical and efficient choice. Community pharmacies in France are individually owned businesses, with no presence of pharmacy chains. As a result, purchasing management is considered a strategic function for the OCPs, as it directly impacts financial performance and cash flow management, making it more difficult to delegate. However, it is observed that this function may occasionally be delegated at a supra-pharmacy level within certain pharmacy networks or groupings. It would therefore be worthwhile to explore whether establishing targets for purchases (volume and discounts) and stock turnover could encourage the delegation of this responsibility to PTs.

Quality assurance management occupies a central position in the preferences of CP respondents, who do not strongly advocate for sharing this task with PTs. It is plausible that CPs do not perceive this activity as particularly contributing to pharmaceutical added value.

Team management, however, is clearly identified as the responsibility of CPs, aligning with their regulatory obligation to supervise PTs and assume responsibility for their professional actions. Interestingly, other studies on task delegation to PTs have highlighted a willingness among PTs to take on management responsibilities for other PTs or non-pharmaceutical staff.^7^^,^^20^ However, the small size of French community pharmacies likely explains why respondents in this study did not consider this activity as relevant.

### 4.5 Pharmaceutical flow activities

Surprisingly, dispensing medication does not rank highest among respondents' preferences for delegation. OCPs rank it 5th, ECPs 6th, and PTs 3rd. This does not suggest that respondents view dispensing as outside the PTs' remit – on the contrary, it remains a core daily task for PTs. The BWS method, designed to ‘force’ an extreme choice and maximize the observable difference in importance between two attributes, guided our approach. We adhered this principle by framing the scenarios not on determining the ‘best’ or ‘worst’ activities overall, but on prompting respondents to clearly differentiate preferences between PTs and CPs. The survey results, therefore, confirm that medication dispensing is already a shared activity between CPs and PTs.

In this survey, we examined the management of supplies for nursing homes, which involves: (i) a logistical process for identifying the institution's needs, procuring the necessary items, and ensuring delivery; and (ii) the dispensing of health products. This activity ranks in the TOP3 for both PTs and CPs, serving as a prime example of the integration between the logistics flow and the standard pharmaceutical flow. These patients typically have scheduled renewals, and their prescriptions remain relatively stable over time. This organizational model could be adapted for other patient groups within the pharmacy setting. *Re*-engineering the pharmacy workflow highlights task delegation towards dispensing as both relevant and efficient: PTs are positioned as assistants for dispensing tasks that do not require immediate or recurring pharmaceutical expertise.^21^ In France, dispensing is predominantly spontaneous: patients visit the pharmacy and wait to receive their medications, with a single operator (CP or PT) performing all dispensing-related tasks. A reorganization of workload, granting PTs greater autonomy within the standard pharmaceutical flow, could support the adoption of medication synchronization in France.^9^^,^^22^ PTs could handle the preparation of treatments in advance and contact patients to gather initial information before dispensing is finalized by a CP upon the patient's visit to the pharmacy.

Rethinking the pharmacy workflow also involves exploring how technicians can contribute to clinical activities.^21^ The results of this survey regarding the screening procedures should be interpreted in the context of conducting Rapid Diagnostic Tests (RDTs). We provided a comprehensive definition of screening, encompassing both common infectious conditions (COVID-19, sore throat, and cystitis) and the identification of patients at risk for chronic diseases such as diabetes and cardiovascular conditions. However, while chronic disease screening remains largely experimental, RDTs are well-established in current practice. It is therefore likely that respondents primarily focused on RDTs when answering this question.

For PT respondents, the issue of conducting tests is intrinsically linked to the question of prescribing for minor ailments: their preference weights for screening and prescribing for minor ailments are nearly identical. In contrast, CP respondents express a preference to delegate the administration of tests while retaining the responsibility for prescribing. These findings may seem surprising, given that in practice, prescribing for minor ailments involves a delegation of task from physicians to CPs – an activity that could even be described as ‘pharmaceutical prescribing’.

How can this divergence in positioning be explained? One potential explanation is that minor ailment prescribing currently pertains to conditions such as cystitis, sore throat, chickenpox, and seasonal allergies – common primary care issues for which pharmacy teams already provide pharmaceutical advice. Moreover, in French practice, this activity is referred to as ‘dispensing under protocol’. This terminology likely reinforces the perception of minor ailment prescribing as a mere extension of dispensing. However, as previously discussed, dispensing is viewed as a shared activity between CPs and PTs. The phrase ‘under protocol’ further suggests a process governed by algorithmic rules – a step-by-step procedure rather than a dynamic decision-making process requiring pharmaceutical expertise. This framing may diminish the recognition of the responsibility and expertise involved in triage and prescribing, overshadowing their clinical significance.

Vaccination prescribing, medication counselling, medication reviews, and the renewal and adjustment of dosages for chronic treatments are activities which attracted the highest preferences as activities within the remits of CPs. These are advanced pharmaceutical care tasks where the pharmaceutical added-value was deemed most significant by the respondents. The hierarchy based on preference weights most likely holds little significance because there appears to have been a dispersion effect in responses to these four activities, with respondents making few real choices. However, in line with the logic of BWS, the survey results indicate that these activities fall within the CP's professional territory, although PTs may not wish to engage themselves in them to the same extent. Indeed, these last seek to spend more time on patient interaction and their integration into cognitive pharmacy services.^23^ PTs could assist in gathering information for medication reviews, thereby facilitating proactive clinical pharmaceutical practice.^7^^,^^20^^,^^24^^,^^25^

### 4.6 RETHINKING supervision

The results of this study indicate a tendency to delegate even more logistical activities to technicians. However, task reorganization must carefully consider the skill set of PTs as well as their interest in pharmaceutical care.^5^^,^^7^^,^^20^^,^^21^ It is not about confining them to being specialists solely in the logistics of the medication use system.

Rethinking the pharmaceutical workflow requires reconsidering how PTs are supervised. In France, current supervision is typically limited to an asynchronous double-check (a pharmacist reviews dispensation made by the team at specific times during the day) or, in some rare cases, real-time oversight.

The pharmaceutical workflow is also heavily reliant on prescription dispensing. This survey examined the activity of medication dispensing, specifying it with the term ‘autonomously.’ At present, PTs dispense medication under the supervision of a CP. A potential reorganization of the workload could involve dividing the pharmaceutical workflow into two distinct categories: a standard, recurrent pharmaceutical flow requiring minimal security or therapeutic optimization, and a specialized pharmaceutical flow necessitating advanced pharmaceutical expertise. The standard flow could be shared between PTs and CPs, depending on workload and available human resources,^21^ with PTs operating under supervised autonomy. The specialized flow, on the other hand, would remain the exclusive responsibility of CPs. Separating the standard pharmaceutical flow from the specialized pharmaceutical flow further involves reflecting on the identification and categorization of patients at risk of therapy-related issues.^26^

Expanding PT responsibilities to include more logistical tasks, involvement in testing, and information gathering (e.g., for medication reviews and disease management) does not align well with their initial training. This training typically focuses on basic pharmacology and on compounding, with minimal to no emphasis on management skills.

Understanding the preferences of CPs and PTs regarding their daily tasks is critical for optimizing pharmaceutical care delivery. By identifying priority activities for each professional group, pharmacy managers can more effectively allocate resources and streamline workflows, ultimately improving patient care outcomes.

Future research should focus on strategies to foster collaboration between CPs and PTs, aiming to maximize the impact of pharmacy services on patient health. Enhanced collaboration could also lead to reduced stress for pharmacists, allowing them to find greater fulfilment in their roles, while technicians could experience a stronger sense of responsibility for ensuring safe and efficient medication distribution.^5^^,^^20^ Empowering technicians and recognizing their contributions is crucial to achieving this balance and enhancing the overall efficiency of pharmacy practice.

The following are the supplementary data related to this article.

**Supplementary Fig. S1:**
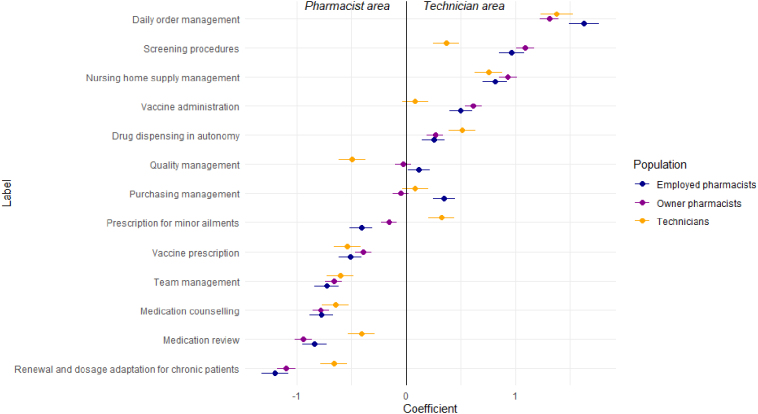

## Funding statement

This research received no specific grant from any funding agency in the public, commercial, or not-for-profit sectors.

## Author contribution

Jean-Didier Bardet: formulates the research question, designs the study, carries it out, analyses the data and writes the article.

Jérôme Combe: interprets the data and writes the article.

Arnaud Tanty: interprets the data and writes the article.

Perrine Louvier: designs the study, carries it out, analyses and interprets the data.

Mathieu Granjon: designs the study, carries it out, analyses and interprets the data.

Benoît Allenet: interprets the data and writes the article.

## CRediT authorship contribution statement

**Jean-Didier Bardet:** Writing – review & editing, Writing – original draft, Validation, Supervision, Software, Methodology, Formal analysis, Data curation, Conceptualization. **Jérôme Combe:** Writing – original draft, Validation. **Arnaud Tanty:** Writing – original draft, Validation. **Perrine Louvier:** Validation, Project administration, Methodology, Formal analysis, Conceptualization. **Mathieu Granjon:** Validation, Project administration, Methodology, Formal analysis, Conceptualization. **Benoît Allenet:** Writing – original draft, Validation.

## Declaration of competing interest

The author(s) declare that there are no conflicts of interest.

## Data Availability

The data was collected by the research team. The database will remain accessible for 18 months after publication of the study results.

## References

[1] Allenet B., Roux-Marson C., Juste M., Honoré S. (2021). Lexique de la Pharmacie Clinique 2021. Le Pharmacien Hospitalier et Clinicien.

[2] République Française (2023).

[3] Legendre B. (2020). En 2018, les territoires sous-dotés en médecins généralistes concernent près de 6% de la population. Etudes & Résultats [Internet]. https://drees.solidarites-sante.gouv.fr/sites/default/files/er1144.pdf.

[4] Ordre National des Pharmaciens (2022). Démographie des pharmaciens - Panaroma 2022 [Internet]. https://www.calameo.com/read/0024493959437f8597cf5.

[5] Hohmeier K.C., Desselle S.P. (2003). Exploring the implementation of a novel optimizing care model in the community pharmacy setting. J Am Pharm Assoc.

[6] Barat E., Pouant C., Soubieux A. (2023). Overview of the implementation of pharmaceutical interviews in pharmacies in France: analysis of responses to a questionnaire. Therapie.

[7] Sparkmon W., Barnard M., Rosenthal M., Desselle S., Ballou J.M., Holmes E. (2023). Pharmacy technician efficacies and workforce planning: a consensus building study on expanded pharmacy technician roles. Pharmacy.

[8] République Française (2019).

[9] Patti M., Renfro C.P., Posey R., Wu G., Turner K., Ferreri S.P. (2019). Systematic review of medication synchronization in community pharmacy practice. Res Social Adm Pharm.

[10] Louviere J., Lings I., Islam T., Gudergan S., Flynn T. (2013). An introduction to the application of (case 1) best–worst scaling in marketing research. Int J Res Mark.

[11] Flynn T.N. (2010). Valuing citizen and patient preferences in health: recent developments in three types of best–worst scaling. Expert Rev Pharmacoecon Outcomes Res.

[12] Société Française de Pharmacie Clinique (2014). Référentiel Pharmacie d'officine [Internet]. SFPC. https://sfpc.eu/wp-content/uploads/2020/10/2014010984-referentiel-pharma-officine.pdf.

[13] Démarche Qualité Officine (2023). Référentiel Qualité [Internet]. 1.7. Paris. https://www.demarchequaliteofficine.fr/referentiel.

[14] Fédération des Pharmaciens de France (2023). Attractivité de la branche de la pharmacie d'officine [Attractiveness of the Community Pharmacy Sector] [Internet]. https://www.fspf.fr/attractivite-de-la-branche-de-la-pharmacie-dofficine-2/?utm_source=chatgpt.com.

[15] Davies J.E., Barber N., Taylor D. (2014). What do community pharmacists do?: results from a work sampling study in London. Int J Pharm Pract.

[16] Karia A., Norman R., Robinson S. (2022). Pharmacist’s time spent: space for pharmacy-based interventions and consultation TimE (SPICE)-an observational time and motion study. BMJ Open.

[17] McCann L., Hughes C.M., Adair C.G. (2010). A self-reported work-sampling study in community pharmacy practice: a 2009 update. Pharm World Sci.

[18] Gregório J., Cavaco A.M., Lapão L.V. (2017). How to best manage time interaction with patients? Community pharmacist workload and service provision analysis. Res Social Adm Pharm.

[19] Cavaye D., Lehnbom E.C., Laba T.L., El-Boustani E., Joshi R., Webster R. (2018). Considering pharmacy workflow in the context of Australian community pharmacy: a pilot time and motion study. Res Social Adm Pharm.

[20] Desselle S.P., Wasem V., Woodyard A., Hosseini S., Hohmeier K.C., McKeirnan K.C. (2023). Cultures of support and resilience are associated with certified pharmacy technicians embracing new roles. Res Social Adm Pharm.

[21] Jetha M., Man K.K.C., Abdulla D., Austin Z. (2021). Exploring multi-stakeholder perceptions of practice-related facilitators to optimising the quality of integration of regulated pharmacy technicians in community pharmacy in Ontario: a qualitative study. Int J Pharm Pract.

[22] Rouger L., Perillat-Bottonet V., Bardet J.D. (2024). XXe Congrès de la Société Française de Pharmacie Clinique.

[23] Boughen M., Sutton J., Fenn T., Wright D. (2017). Defining the role of the pharmacy technician and identifying their future role in medicines optimisation. Pharmacy (Basel).

[24] Société Française de Pharmacie Clinique (2023). Préconisations pour la contribution des préparateurs en pharmacie aux actions de pharmacie clinique. Le Pharmacien Clinicien.

[25] Amador-Fernández N., Escaith M., Simi E., Quintana-Bárcena P., Berger J. (2023). Evaluation of an enhanced service for medication review with follow up in Swiss community pharmacies: Pre-post study protocol. Köpke S, editor. PloS One.

[26] Pammett R.T., Blackburn D., Taylor J. (2015). Evaluation of a community pharmacy-based screening questionnaire to identify patients at risk for drug therapy problems. Pharmacotherapy.

